# First evidence of the effectiveness of a field application of RNAi technology in reducing infestation of the mite *Varroa destructor* in the western honey bee (*Apis mellifera*)

**DOI:** 10.1186/s13071-025-06673-7

**Published:** 2025-01-27

**Authors:** Francesca Bortolin, Emanuele Rigato, Sergio Perandin, Anna Granato, Laura Zulian, Caterina Millino, Beniamina Pacchioni, Franco Mutinelli, Giuseppe Fusco

**Affiliations:** 1 Department of Biology, University of Padova, Padova, Italy; 2 Smart Bugs, Ponzano Veneto, Treviso, Italy; 3 Independent Researcher, Montebelluna, Treviso, Italy; 4 National Reference Laboratory for Honey Bee Health, Istituto Zooprofilattico Sperimentale delle Venezie, Legnaro, Padova, Italy

**Keywords:** dsRNA, Pest control, *Varroa destructor*, Honey bee, Biopesticide

## Abstract

**Background:**

The mite *Varroa destructor* is the most serious pest of the western honey bee (*Apis mellifera*) and a major factor in the global decline of colonies. Traditional control methods, such as chemical pesticides, although quick and temporarily effective, leave residues in hive products, harming bees and operators’ health, while promoting pathogen resistance and spread. As a sustainable alternative, RNA interference (RNAi) technology has shown great potential for honey bee pest control in laboratory assays, but evidence of effectiveness in the field has been lacking.

**Methods:**

We investigated the efficacy and feasibility of a RNAi treatment to improve bee health under natural beekeeping conditions by integrating a honey bee diet with a mixture of dsRNA targeting *V. destructor* acetyl-CoA carboxylase, Na^+^/K^+^ ATPase and endochitinase genes.

**Results:**

In treated hives, we observed that the average infestation rate of phoretic *Varroa* mite was reduced by 33% and 42% relative to control bees fed with sucrose and GFP-dsRNA, respectively. The dsRNA treatment did not affect bee survival, and the beekeepers involved in the project found the method manageable in the apiary and non-intrusive to production activities.

**Conclusions:**

Our findings demonstrate the feasibility and effectiveness of RNAi technology in reducing *Varroa* mite infestations under natural rearing conditions. This study supports the potential of RNAi as a promising alternative to chemical pesticides, offering a targeted, efficient and sustainable solution for managing *V. destructor* in honey bee populations.

**Graphical Abstract:**

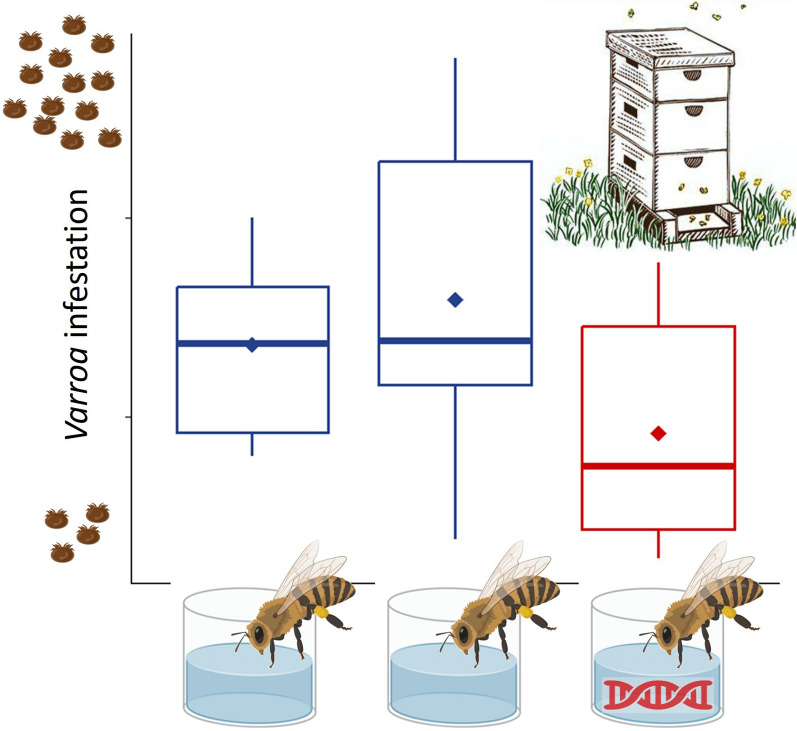

**Supplementary Information:**

The online version contains supplementary material available at 10.1186/s13071-025-06673-7.

## Background

The ectoparasitic mite *Varroa destructor* is the most serious pest of the western honey bee, *Apis mellifera*, worldwide. No other pathogen or parasite has had a comparable impact on this species, in part because *V. destructor* only recently adapted from its original host, the Asian honey bee *Apis cerana*, to exploit a new host with very limited innate defences. Contrary to what occurs in *A. cerana* colonies, in *A. mellifera* the parasite infests both the drone brood and the more persistent worker brood, leading to higher infestation levels [1]. The mite feeds on fat bodies of adult bees during its dispersing phase and primarily on haemolymph of bee pre-imaginal stages during the reproductive phase [2]. This causes several injuries, such as reduction of body weight in hatching bees, a deficit in sperm production in drones, alteration of flying, homing and orientation abilities in foragers, and downregulation of honey bee’s immune response [3]. *Varroa destructor* is also a viral reservoir and the main transmitter of some honey bee-associated viruses like Deformed wing virus (DWV). Although bee viruses usually persist as unapparent infections, under certain stressful conditions they can dramatically cause serious or lethal disease in individual bees or the collapse of entire colonies (e.g. [4]).

In addition, several studies have shown that *V. destructor* can interact with other biotic and abiotic stressors, such as environmental factors, other parasites and pesticides, leading to an even more serious impact on honey bee health [5–7]. Therefore, *V. destructor* is considered the major driver of honey bee colony decline around the world, with important economic losses in the beekeeping sector, due to both the lack of production and the increase in the costs necessary for treatments [8]. If mite populations remain undetected and untreated, infested honey bee colonies usually collapse within 1 to 3 years [1, 9].

To treat mite infestation, beekeepers have relied mainly on synthetic acaricides, such as formamidines, organophosphates and pyrethroids, because they are generally easy and fast to use and generally very effective. However, their effectiveness has decreased in recently because of their extensive use, resulting in the evolution of the mite’s resistance in apiaries from several countries [10, 11]. Moreover, these acaricides generate residues that accumulate in beeswax, bee bread and honey, and they can be transferred to brood and adult honey bees, with negative effects on the colony's health [12–14].

Because of the adverse impact that synthetic acaricides have on bees and bee products worldwide, beekeepers are increasingly using non-hard chemical control methods, like essential oils and organic acids, which are usually less efficient compared with synthetic acaricide treatments but still effectively able to control mite populations [15]. Organic acids are naturally found in bee products and have a lower risk of triggering resistance in mites [16] but can nonetheless have some negative effects on bees, such as decreasing worker populations, increasing capped brood removal or decreasing drone sperm quality [17].

As set out in the “Farm-to-fork” initiative, a strategy aiming at accelerating the European transition towards a sustainable food system, the European Commission has adopted measures to reduce by 50% the overall use of synthetic pesticides and the resulting risk by 2030, at the same time promoting greater use of alternative methods of protection from parasites and diseases (European Green Deal 2020). Therefore, it is crucial to develop alternative approaches to treating *V. destructor* that do not generate resistant populations of mites and are safe for bees, bee products, beekeepers and the environment.

Utilisation of RNA interference (RNAi), an intracellular mechanism of sequence-specific gene silencing conserved across eukaryotes, has been proposed as a targeted and sustainable pest-control strategy, in particular in agriculture [18]. RNAi-based technologies for pathogen or pest control exploit this pathway to suppress the expression of specific gene transcripts through the delivery of sequence-specific dsRNA complementary to mRNA transcripts that encode for proteins important for the survival or reproduction of the target organism [19]. dsRNAs are emerging as a potential alternative to synthetic pesticides, because their sequence-dependent mode of action makes them more selective, efficient and flexible compared to other conventional agrochemicals. Besides, dsRNAs generally have limited environmental persistence in soil, sediment and water and do not affect human health [20, 21].

Over the last decade, research has explored the efficacy of RNAi in the control of several common honey bee pathogens and parasites including viruses like DWV [22, 23], Israeli acute paralysis virus (IAPV; [24, 25]) and Sacbrood virus (SBV; [26, 27]), the microsporidian *Vairimorpha ceranae* (formerly, *Nosema ceranae*; [28, 29]) and the small hive beetle *Aethina tumida* [30].

Laboratory studies on *V. destructor* have shown that injection of dsRNA or soaking of the mites into a dsRNA solution can result in significant gene silencing, although the efficacy depends on the target gene [31–33]. As an alternative, feeding bees with a syrup supplemented with dsRNAs that target mite genes resulted in effective uptake by mites, in turn reducing their survival [34] or fertility [35]. Recently, a symbiotic bacterium from honey bee gut was engineered to repeatedly produce dsRNA against genes essential for *V. destructor* metabolism and was successfully fed to the bees. Mites on bees nourished with the engineered bacteria had a reduced survival rate compared with mites feeding on control bees [23].

These promising results were all obtained under controlled conditions, but no field trials were carried out. In contrast to laboratory experiments, where dsRNA effects are tested in isolation and on small scales, open field environments present physical and biological parameters which are largely unpredictable and highly dynamic.

We investigated the efficiency and feasibility of an RNAi treatment to improve bee health under natural beekeeping management. We integrated western honey bee diet with dsRNAs against *V. destructor* gene sequences to determine whether this reduces the parasite load in field conditions.

This research is part of the project “BeeOShield”, funded by Rural Development Program for Veneto region 2014–2020 (Measure 16), a European Union instrument that allows member states, and in this case the individual Italian regions, to support increasing innovation in agriculture and forestry-related activities. Specifically, projects funded under Measure16 are expected to be experimental research aimed at an immediate follow-up on agriculture and forestry practice to be developed in cooperation with stakeholders. This is motivated by the need to fill the counterproductive lack of effective interactions between researchers and practitioners in this field, especially in Europe.

In this context, we developed the project together with the beekeepers managing the apiaries involved in the trial, adapting the experimental protocol to their production needs, involving them directly in the administration of the dsRNA and gathering their feedback and suggestions. This approach, despite having imposed some limitations on data collection (like, for instance,  allowing direct measurement of phoretic infestation levels only, see below), allowed us to evaluate the feasibility of the transition from laboratory to field of the RNAi-based technology on *Varroa* mite control. The positive results of the present work, while preliminary, encourage further developing and enhancing these techniques of honey bee management.

## Methods

### Target gene selection and dsRNA synthesis

Genes to be silenced were chosen among the targets of acaricide compounds that reduce survival of mites by inhibiting gene function.

Acetyl-CoA-carboxylase (ACC) is an enzyme that plays a fundamental role in fatty acid metabolism. The tetronic/tetramic acid family of acaricides inhibit ACC binding to the carboxyltransferase domain, thus interfering with the biosynthesis of lipids in insects and mites [36].

Na^+^/K^+^ ATPase is a membrane-bound enzyme responsible for ion transport which has an important role in the regulation of membrane permeability and osmotic balance. This ATPase is the target of some defensive compounds produced by plants, such as pyrethrins and cardiac glycosides, which exhibit strong toxicity against insects and mites [37, 38].

Chitinases (CHITs) are enzymes involved in chitin degradation and reconstruction during the process of arthropod moulting. Some acaricides such as diflubenzuron and scopoletin interfere with the expression of chitinase genes and thus prevent mites from undergoing normal growth and development [39].

A 248-bp dsRNA (VdACC-dsRNA) was designed in the carboxyltransferase domain from the *V. destructor* acetyl-CoA carboxylase mRNA sequence (XM_022805405); a 249-bp dsRNA (VdATPase-dsRNA) was designed from the *V. destructor* Na^+^/K^+^ ATPase mRNA sequence (XM_022791887) and a 211-bp dsRNA (VdChit-dsRNA) was designed partially in the glycosyl hydrolase 18 conserved domain from the *V. destructor* endochitinase mRNA sequence (XM_022796590). All sequences were retrieved from the NCBI database. Since using RNAi for mite control requires that it does not negatively affect honey bee health, we compared the sequences of the three candidate dsRNAs with the *A. mellifera* genome to prevent off-target bee gene silencing. The dsRNA for the green fluorescent protein (GFP-dsRNA, 432 bp), which served as a negative control, was taken from previous studies [22, 24]. dsRNA sequences are available in Additional file 1: Text S1.

The large quantity of dsRNA was synthesized in vitro by AgroRNA (Genolution, Seoul, South Korea), shipped in distilled water at ambient temperature and kept at –20 °C until use.

### Effectiveness of dsRNA treatment in the laboratory

#### Administration of dsRNA by soaking mites

*Varroa* mites were collected from highly infested hives of one of the apiaries (TV6; Additional file 1: Table S1). Adult mites were dislodged from adult honey bees with powdered sugar and rinsed with water, and 30 mites were randomly assigned to each of four treatment groups: (i) VdACC-dsRNA, (ii) VdATPase-dsRNA, (iii) VdChit-dsRNA and (iv) control group, with five biological replicates for each one. They were placed in 500 μl microfuge tubes containing 2.5 μg/μl dsRNA (specific of each group) in 0.9% NaCl solution or saline solution only for controls. Mites stayed immersed at 4 °C for 14 h before being removed from the solution, dried and placed separately in Petri dishes for each group and replica, where they were fed on same-age bee larvae at 27 °C and 70% relative humidity. To evaluate the level of target gene expression, surviving mites were sampled from each experimental group at 48 h after the end of the treatment and stored at –20 °C.

#### RNA isolation and cDNA synthesis

To validate RNAi in soaked mites, total RNA was extracted from a pool of 15–20 mites for each treatment group and replicate. Biological replicates were extracted and analysed separately. Mites were homogenized in 350 μl lysis buffer RA1 (Machery Nagel, Germany) with 5-mm stainless steel beads in a bead mill homogenizer (Tissue Lyser II; Qiagen, Germany) for 2 min at 30 Hz. After centrifugation (5 min, 10,000 × g), the supernatant was used for RNA isolation with Nucleo Spin RNA kit (Macherey Nagel). RNA was eluted into 60 μl RNase-free H_2_O. After centrifugation, the eluate was applied once more onto the column for a second elution. The yield and purity of the extracted RNA (260/280 and 260/230 nm absorbance ratios) were assessed with a Nanodrop N1000 spectrophotometer (NanoDrop Technologies Inc., USA).

First-strand cDNA was synthetized from 1 µg total RNA using the SuperScript III First-Strand Synthesis System for RT-PCR kit (Invitrogen, USA) following the manufacturer’s protocol.

#### Primer design and qPCR analysis

The expression of target genes in *Varroa* mite was quantified with qPCR using a 7500 Real Time PCR System (Applied Biosystem, USA) by Microarray Service (Department of Biology, University of Padova). The employed primers are listed in Table 1.

**Table 1:** Primer sequences used in this study

Gene	Primer	Primer sequence 5’-3’	Length (bp)	Reference
*Acetyl-CoA-carboxylase*	VdACC_F	CATTGAACTCTGTAAACGC	79	New, designed on sequence XM_022805405
	VdACC_R	TCCTTGCCGATGATATTC		New, designed on sequence XM_022805405
*Na*^+^*/K*^+^ *ATPase*	VdATPase_F	GTGCGGACAACTGACAAC	72	New, designed on sequence XM_022791887
	VdATPase_R	AAACACGACGAACGAACAC		New, designed on sequence XM_022791887
*Endochitinase*	VdChit_F	TTGACGATTGGGGTTATG	138	New, designed on sequence XM_022796590
	VdChit_R	GATTGTCTTTGCTACCTAACG		New, designed on sequence XM_022796590
*Actin *(reference gene)	VdAct_F	TCATCGGAATGGAGTCAT	105	New, designed on sequence AB242568
	VdAct_R	CAGAGAGAACGGTGTTAGC		New, designed on sequence AB242568
*NADH dehydrogenase *(reference gene)	VdNADH_F	CACGGTCGAAGAAGAAATGA	96	New, designed on sequence XM_022804344
	VdNADH_R	ATCACGCACAGCAGGTTATC		Ref. [59]
*Succinate dehydrogenase* (reference gene)	VdSDHA_F	TCCAATCCTTCCAACTGTCC	98	Ref. [59]
	VdSDHA_R	CGACCTTATCCTGACCTTGTG		New, designed on sequence XM_022806549

Primers’ specificity and efficiency were assessed by qPCR on cDNA from a pool of mites (RNA extraction and reverse transcription protocol as above) using twofold serial dilutions of cDNA ranging from 50 to 6.25 ng and a final primer concentration of 1 µM. Each dilution was analysed in triplicate. Actin, NADH dehydrogenase and succinate dehydrogenase were tested as reference genes.

qPCR assays were performed in a volume of 10 µl containing 5 µl 5× PowerUp SYBR Green Master Mix (Applied Biosystems), 50 ng cDNA and primers (1 µM final concentration). Reactions were performed in triplicate (technical replicates) using the following protocol: preincubation (50 °C for 2 min, 95 °C for 2 min), 40 cycles of 2-step amplification (95 °C for 20 s, 60 °C for 1 min) and a melting step (95 °C for 15 s, 60 °C for 1 min and 95 °C for 15 s, with a ramp rate of 4.4 °C/s for heating up and 3.4 °C/s for cooling down).

The relative gene expression levels in each treatment group were determined by using the comparative delta Ct (threshold cycle number) method (2^−ΔΔCt^). The difference between the Ct values (ΔCt) of the target gene and the reference gene was calculated for each technical replicate, as well as the 2^−ΔCt^ value. The mean normalized gene expression value from the three technical replicates was then computed.

### Survival analysis

The possible effects of dsRNA intake on bee survival were tested in six bioassay cages (minihives, 12 × 15x7 cm), containing 31–35 bees each. Adult bees collected from a single hive (in TV6 apiary; Additional file 1: Table S1) were placed in minihives and maintained at 27 °C and 55% relative humidity for 21 days. In three minihives of the treatment group, bees were fed daily with a mixture of VdACC-dsRNA, VdATPase-dsRNA and VdChit-dsRNA in 2 ml 60% sucrose solution for the first 7 days, at a dose of 1 μg of each dsRNA per bee per day, and with sucrose solution in thereafter. In three other minihives of the control group, bees were fed only with sucrose solution.

Each minihive was inspected daily, recording the numbers of dead individuals, which were contextually removed from the cage.

### Effectiveness of dsRNA treatment in the field

The effectiveness of dsRNA under natural beekeeping conditions was planned to be tested in 50 colonies across 5 apiaries, located in the plain of Veneto region (northeastern Italy) and belonging to different beekeepers (Additional file 1: Table S1). In each apiary, we selected ten hives (Dadant Blatt type with 10 frames) with honey bee colonies of approximately equal strength. The strength of each bee colony was evaluated at the beginning and end of the experiment (day 1 and day 37 of the experiment, respectively), while the overall condition of hives, including a control for main bee diseases, was checked at day 1, day 16 and day 37 of the experiment. The evaluation of the strength of each bee colony encompassed various parameters, such as the number of adult bees, the quantity of the brood and the amount of honey and pollen reserves. Following the Liebefeld method, size of the adult bee population was estimated by measuring the area covered by bees on each frame of the hive and converting it into the number of bees using established conversion factors [40, 41]. The same method was applied to quantify the amount of the brood, distinguishing between open and operculated cells, as well as to assess honey and pollen reserves. Inspections were made at approximately the same time of the day in all apiaries to limit daily variation in the number of bees in the hive. Visual inspection of the colony allowed assessing the presence of abnormal behaviours and clinical signs related to brood diseases, such as Chalkbrood, American foulbrood, European foulbrood and adult pathologies, such as Deformed wing virus (DWV), Acute bee paralysis virus (ABPV) and Chronic bee paralysis virus (CBPV). Nosemosis caused by the microsporidia *Vairimorpha ceranae* (formerly, *Nosema ceranae*) was quantified in a sample of 60 bees for each hive, following the protocol described in [42]. External factors possibly affecting the colonies, such as climatic events, food scarcity or chemical treatments carried out nearby the hives, were annotated as well. The authorization to conduct clinical trials of dsRNA on animals was granted by Italian Ministry of Health.

Data collection and *Varroa* sampling were performed by a single operator (SP), whereas dsRNA administration was carried out by the beekeeper managing the apiary. To meet production needs, the experiment was scheduled away from the harvesting of honey and from the subsequent traditional treatment with acaricides, which was carried out at the end of July. Before treatment with the oxalic acid drugs, the queen bees were caged to prevent egg laying for 24 days.

The experiment was conducted in 2022 from September 20 to November 9. In each apiary, hives were randomly assigned to three treatment groups. In the *Varroa* dsRNA-treated group (dsT), bees were fed with a mixture of VdACC-dsRNA, VdATPase-dsRNA and VdChit-dsRNA at a dose of 0.8 µg of each dsRNA per bee per day, while in the GFP control group (gfpC) bees were fed with GFP-dsRNA at a dose of 1 µg per bee per day (following [34] protocol). In these two groups, dsRNAs were mixed with 200 ml 60% sucrose solution per hive and supplied seven times (once every 3 days). Bees in the sucrose control group (sucC) were fed with only 60% sucrose solution. In all groups, bees were served using a small 210-ml vacuum feeder, a container with small holes drilled on the lid. The feeder was turned over the hole in the honeycomb cover so that the bees could suck the syrup from the holes in the lid of the container.

The infestation rate of phoretic *Varroa* mites was evaluated using the powdered sugar shaking method [43, 44]. A preliminary check to control that all the selected hives presented a similar level of infestation was conducted in advance of the experiment, at the end of August. Mite infestation level was then assessed on the 1st day of the experiment before the first dsRNA administration (day 1) and the 16th day after the end of dsRNA administration (day 37 of the experiment). In the experiment, three replicates of powdered sugar shaking were performed for each hive (on approximately 300 adult bees each) for a more accurate estimate of the colony infestation level [44].

The effectiveness of dsRNA treatment was evaluated based on the variation in the level of phoretic infestation between day 1 and day 37.

### Statistical software

Statistical analyses were carried out with R (ver. 2023.09.1 + 494; http://www.R-project.org). Accessory calculations were performed in Microsoft Excel (ver. 2310).

## Results

### Effectiveness of dsRNA treatment in the laboratory

Of the three genes tested as a reference, succinate dehydrogenase (*SDHA*) proved the most suitable because it generated a Ct value comparable to those of the target genes, a sharp peak by melting curve analysis and absence of non-specific products or primer-dimer artefacts.

**Fig. 1:**
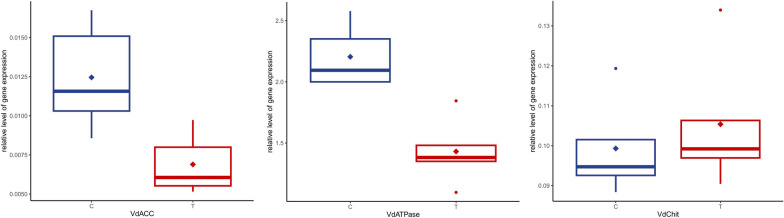
Relative levels of gene expression for *Varroa destructor* acetyl-CoA carboxylase (VdACC), *V. destructor* Na^+^/K^+^ ATPase (VdATPase) and *V. destructor endochitinase* (VdChit) relative to *SDHA* expression level in control (C) and treated (T) groups. Boxes represent the interquartile interval, with median (transverse line) and mean (diamond); vertical lines are ranges of variation, to the exclusion of outliers (dots). Neither mean nor median differences are statistically significant for the gene VdChit

Soaking mites in dsRNA solutions induced a statistically significant gene silencing in two out of three target genes. Average gene expression was significantly reduced relative to control by 45% for the *V. destructor* acetyl-CoA carboxylase gene and by 35% for the *V. destructor* Na^+^/K^+^ ATPase gene (one-tailed Student’s t-tests, *n*_C_ = 5, *n*_T_ = 5, *df* = 8; *t* = 3.19, *P* = 0.0064 and *t* = 4.66, *P* = 0.0008, respectively), but it was not significantly different for the *V. destructor* endochitinase gene (Fig. 1).

### Survival analysis

**Fig. 2:**
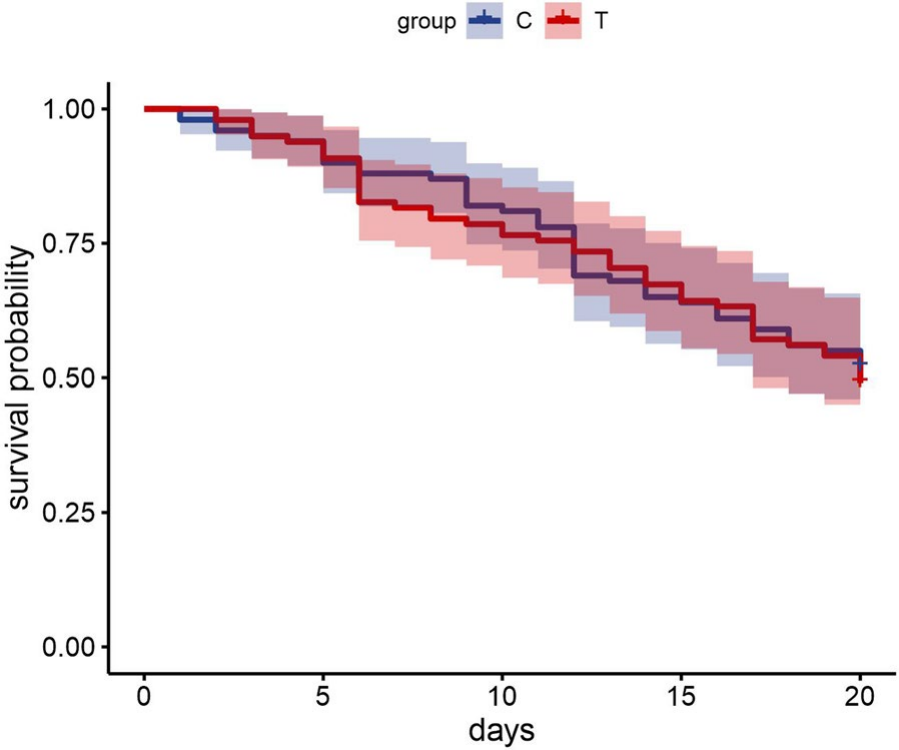
Kaplan-Meier survival probability curves of bees fed with the dsRNA mixture (T) compared with bees fed only with sucrose solution (C). Shaded areas are 95% confidence intervals

The assay, performed to establish whether RNAi targeting *Varroa* genes produce unexpected effects on honey bee survival, showed that the oral administration of dsRNA mixture was safe for the insect. Kaplan-Meier survival probability curves of treated and control groups (for our data, equal to the complement of empirical cumulative distribution functions) did not show significant differences (log-rank test, *n*_C_ = 100, *n*_T_ = 98, *df* = 1, χ^2^ = 0.11, *P* = 0.73) (Fig. 2).

We did not perform an analogous test on the bee larvae, but the post-experiment evaluation of the strength of the colonies supports the safety of dsRNA administration for pre-imaginal developmental stages as well. At the end of the experiment (day 37), we did not record any significant differences in the strength of the colonies between treated and control groups for either the brood (one-way ANOVA, *df* = 36, *F* = 0.24, *P* = 0.79) or the adults (one-way ANOVA, *df* = 36, *F* = 1.18, *P* = 0.32).

### Effectiveness of dsRNA treatment in the field

Data on mite infestation collected from 37 hives managed by beekeepers were included in the following analyses. Of the 50 colonies of the project, we excluded two colonies that collapsed during the experiment (one hive robbed and another orphan) and all the ten colonies of one apiary seriously affected by a mosquito treatment conducted very close to hives. We also dropped one more hive because this was the only one exhibiting an anomalous dynamic of the infestation: a neat decrease in *Varroa* load rather than the expected autumn increase, which we were unable to explain based on available supplementary information on the apiary (Additional file 1: Table S1). Most hives showed no signs of diseases during the whole experiment, except for a *V. ceranae* infection in five hives of one apiary (TV2) at day 1 of the experiment, of which only one was still infected at the end of the trial (day 37).

**Fig. 3:**
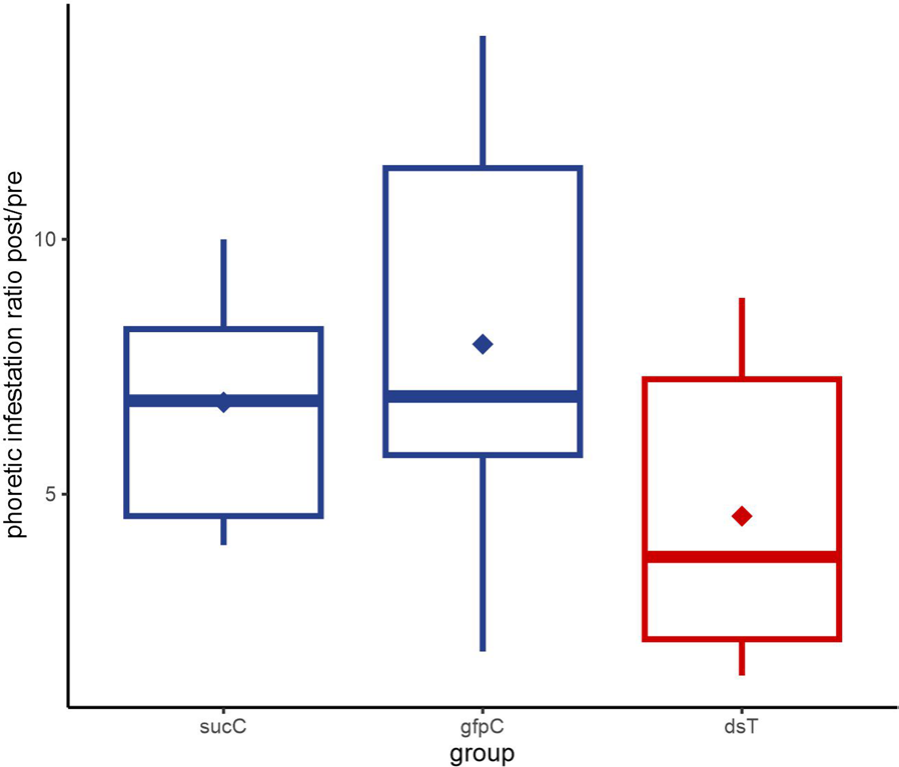
Variation in the level of phoretic *Varroa* infestation (IR_post/pre_) between day 1 and day 37 of the experiment for the three treatment groups: sucrose control (sucC), GFP control (gfpC) and dsRNA treatment (dsT). Boxes represent the interquartile interval, with median (transverse line) and mean (diamond); vertical lines are ranges of variation. Both mean and median of IR_post/pre_ in dsT group were significantly lower than in both control groups

Variation in the level of phoretic infestation was computed as the post/pre-infestation ratio (IR_post/pre_) between day 1 and day 37 of the experiment. The mean IR_post/pre_ with mix dsRNA treatment (dsT) was significantly smaller of both the GFP control (gfpC) by 42% (one-tailed Student’s t-test, *n*_C_ = 9, *n*_T_ = 19, *df* = 26; *t* = 2.60, *P* = 0.0077) and sucrose control (sucC) by 33% (one-tailed Student’s t-tests, *n*_C_ = 9, *n*_T_ = 19, *df* = 26, *t* = 2.05, *P* = 0.0255). The same was observed for the median IR_post/pre_, which was reduced by 46% and 45%, respectively (Mann-Whitney U tests, *U* = 45.0, *P* = 0.0245 and *U* = 46.5, *P* = 0.0291) (Fig. 3).

## Discussion

Several studies on insect pest control have shown that the efficacy of RNAi treatments varies extensively not only among organisms but also within the same species when different genes, tissues and developmental stages are targeted [45]. For instance, a review study on RNAi experiments in lepidopterans reported that only 38% of 130 analysed genes were silenced at high levels, while 48% and 14% of the genes failed to be silenced or were silenced at low levels, respectively [46].

In our experiments, the soaking of adult *Varroa* mites in a dsRNA solution was effective in reducing acetyl-CoA-carboxylase and Na^+^/K^+^ ATPase gene expression, but failed to silence *V. destructor* endochitinase. A possible explanation is that this gene, like others, is regulated by post-translational mechanisms; thus, feedback mechanisms of regulation might readily counteract depletion of mRNA levels with higher rates of transcription [47]. Another explanation could be that the developmental stage of *Varroa* at the time of the treatment did not coincide with the time of most intense expression of the target gene. In fact, it has been shown in other organisms that the effectiveness of gene knockdown critically depends on their developmental stage at the time of dsRNA administration, being greatest when target genes are more intensively expressed [48, 49]. Chitinases are enzymes that play an important role in regulating the moulting process, and in the mites *Panonychus citri* and *Tetranychus cinnabarinus*, they are more abundant in larval and nymphal stages than in adults [39, 50]. A study of the expression profile of *V. destructor* endochitinase and its silencing with dsRNA during different developmental stages of *Varroa* mites could possibly reveal an association between the efficacy of RNAi and the temporal expression profile of the gene in this species, but currently this remains a conjecture.

In any case, in planning RNAi-based mite control strategies, it is important to implement a multi-target approach. Following the selection of a panel of suitable target genes involved in different physiological processes, like metabolism, feeding and reproduction of the target organism (whose knockout does not result in detrimental effects on associated non-target organisms), simultaneous multiple-gene silencing obviously has the potential to increase the impact on the target organism while levelling out the effectiveness (hardly predictable) of the single dsRNAs. In addition, the design of dsRNAs simultaneously targeting multiple genes, or multiple portions of the same gene, can hinder the capacity of the target organism to develop resistance, through mutations in the mRNA targeted by a single dsRNA sequence.

The possibility of effectively silencing *V. destructor* genes through the ingestion of dsRNAs previously ingested by honey bees has been proved in controlled laboratory conditions [23, 34, 35]. In this study, we aimed at further exploring the possibility of using RNAi techniques to control the mite load in *A. mellifera* colonies under field conditions. Bees fed with a sucrose syrup containing a mixture of dsRNAs targeting *V. destructor* genes showed a reduced increase of *Varroa* infestation compared with bees fed with sucrose or GFP-dsRNA. This difference in mite load increase among groups was recorded 16 days after the last dsRNA administration. This seemed to be a sensible time, allowing these molecules to spread throughout the hives and impact the mites. In fact, dsRNAs ingested with food are horizontally transferred among adult bees via trophallaxis and across generations with royal jelly [51]. Next, dsRNAs are assimilated by female mites during the time they are immersed in the royal jelly or while feeding on developing bees. Our results show that this complex, cross-species dsRNA route is also effective in field conditions, provided some specific measures to prevent the degradation of the molecules are taken. These include the proper storage temperature for dsRNA until the application, reduction of any contamination in handling solutions, daily check of syrup consumption and thermal insulation of hive roof.

Most available chemical control methods for this mite, including synthetic chemicals, essential oil components and widely used organic acids, only impact phoretic mites [1]. Mites concealed in broods are more difficult to get rid of because the wax capping on the brood cells protects them while they reproduce underneath. Therefore, the hardest time to control mite populations with traditional methods is when the colony has high brood numbers. In contrast, the systemic spread of dsRNA through the colony by horizontal transfer from treated larvae to *Varroa* inside the brood cell can also affect the reproductive phase of the mite. This approach can help to reduce the likelihood of high mite infestation levels exactly when it is more difficult to effectively control mite populations with traditional acaricides.

Under temperate climatic conditions, *Varroa* treatments must be performed before the production of long-lived winter bees because worker bees parasitized during development have a reduced life span and will presumably not survive until spring [52]. An effective *Varroa* control in autumn is crucial for successful overwintering of honey bee colonies, and although dsRNA treatment does not lead to a complete eradication of *Varroa*, it can contribute to keeping the autumn infestation levels below the threshold indicated as an acceptable colony loss rate in winter [53].

Worker bees fed dsRNAs by the oral route showed no survival differences compared to control bees fed with sucrose. This is in accordance with other studies which recorded a generalized insensitivity of honey bees to environmental dsRNA [54–56].

Beekeepers involved in our project pointed out that this method is easily manageable in the apiary, and it does not interfere with production activities. In addition, differently from acaricides, dsRNAs do not require either protective equipment during handling or particular skills for their application. Although data on the impact of dsRNA application on the environment and user and consumer health are still limited, a risk assessment by the European Food Safety Authority (EFSA) classified RNA biopesticides as safe because of the low risk that sprayable RNAs pose for animals/humans [57]. dsRNA residues in honey bee products have not been evaluated, but even if they were present, it is assumed that their oral uptake would carry a low risk for interference with gene expression in humans because of the very effective gastric barrier in vertebrates [21, 58] and the expected absence of a match with human gene sequences.

Further experiments under controlled conditions are needed to understand the effect of dsRNA on the reproductive phase of *V. destructor*. Additionally, it is crucial to set up the duration, adequate administration frequency and best seasonal schedule of the treatments to induce a more effective reduction of mite infestation.

## Conclusions

The “BeeOShield” project involved apiaries located in different areas that experienced various environmental and management conditions. This approach compelled us to exclude hives from the analysis where unpredictable occurrences affected the results of RNAi treatment, resulting in a low sample size. In addition, since we used honey bee colonies not intended for research purposes, we had temporal constraints on the experimental design and could not perform any invasive measures to evaluate mites inside brood cells so as not to interfere with production needs. Despite these limitations, the setting of this trial under natural rearing conditions and the interactions with beekeepers who managed apiaries allowed us to assess that RNAi-based technology to sustain honey bee health is feasible in a productive context. Our preliminary results on containment of mite infestation support the candidature of dsRNAs as a promising alternative to conventional chemical pesticides. This technology can support good beekeeping practices, helping to reach superior long-term mite control using a more selective and sustainable approach to benefit bees, humans and the environment.

## Supplementary Information

Supplementary Material 1.Text S1. dsRNA sequences used in this study. Table S1. Apiaries and number of hives involved in the experiment.

## Data Availability

Data supporting the findings of this study are available from the corresponding author on request.
