# Correction: First evidence of the effectiveness of a field application of RNAi technology in reducing infestation of the mite *Varroa* destructor in the western honey bee (*Apis mellifera*)

**DOI:** 10.1186/s13071-025-06778-z

**Published:** 2025-04-02

**Authors:** Francesca Bortolin, Emanuele Rigato, Sergio Perandin, Anna Granato, Laura Zulian, Caterina Millino, Beniamina Pacchioni, Franco Mutinelli, Giuseppe Fusco

**Affiliations:** 1 Department of Biology, University of Padova, Padua, Italy; 2 Smart Bugs, Ponzano Veneto, Treviso, Italy; 3 Montebelluna, Treviso, Italy; 4 National Reference Laboratory for Honey Bee Health, Istituto Zooprofilattico Sperimentale delle Venezie, Legnaro, Padua, Italy

**Correction: Parasites & Vectors (2025) 18:28** 10.1186/s13071-025-06673-7

Following publication of the original article [1], it came to the author's attention that there was an error in the Methods section: in the subsection 'Administration of dsRNA by soaking mites', it (incorrectly) said 'Mites stayed immersed at 14 °C' in place of 'Mites stayed immersed at 4 °C'. The article has since been corrected. The authors thank you for reading this erratum and apologize for any inconvenience caused.
