# Supplementary material for: First evidence of the effectiveness of a field application of RNAi technology in reducing infestation of the mite Varroa destructor in the western honey bee (Apis mellifera)
Source: Parasit Vectors. 2025 Jan 27;18:28. doi: 10.1186/s13071-025-06673-7 (PMC11771053; doi:10.1186/s13071-025-06673-7)
Supplement: Supplementary file 1 — Supplementary Material 1.Text S1. dsRNA sequences used in this study. Table S1. Apiaries and number of hives involved in the experiment. [file 13071_2025_6673_MOESM1_ESM.pdf]

Supplementary information

Text S1
dsRNA sequences used in this study

VdACC-dsRNA (248bp)
TTACGGTACACGACGATCTTGAGGGTTGTTACACAATGTTGAAGTGGCTATCGTATATGCCTCGAATTAAG
GGAGGTGACCTGCCAATCCTCGAGAGTATGGACCCATTCGAGCGAGATGTTGTGTTTACGCCGACGAAGG
CCCCGTATGACCCGCGATGGCTACTCGCTGGACGCGAGAGTCCCAACTTACCTGGCTTCTGGGAGGACGG
ATTCTTTGACAGAGGTAGTTTCTCTGAAATTATGGCT

VdChit-dsRNA (211bp)
TGATGGCAATTGACCCAAATTTGGACATCGAGAAAAACGGTTATAAACGCTTCAACGATCTTAAGAGAAA
GCACTCGAATCTCAAAACAATTCTAGCCATTGGTGGCTGGGATGAAGGTGGCCAAAAATACTCGGACATG
GTAAGCTCGAAAGAACGGCGTGCAACATTTGTGCAGTCTGCAGTCAAGTGGGTCAAGGAACATGATTTCG
A

VdATPase-dsRNA (249bp)
GCATTCTGGACGGTTATGCTGGTCATCTTCTATCAGACACTCGATGCCTTCCAGCCAAAGTGGACCCTGGA
CGCTAGTCTCATTGGCACTGTACCGGGATTAGGCTTCAGGCCACGCCCACCGCTGTCTAACATCGACTCAA
CACTCATCTATTTCAAGGCGGGAGGAAGCGACAGAGATTCATACAAGCACTGGGTGAAGGACCTCGATG
ATTTTATTGAGAAGTATCGCGATGCAGGCAACACCGGG

GFP-dsRNA (432bp)
GCCAACACTTGTCACTACTTTCTCTTATGGTGTTCAATGCTTTTCAAGATACCCAGATCATATGAAACGGC
ATGACTTTTTCAAGAGTGCCATGCCCGAAGGTTATGTACAGGAAAGAACTATATTTTTCAAAGATGACGG
GAACTACAAGACACGTGCTGAAGTCAAGTTTGAAGGTGATACCCTTGTTAATAGAATCGAGTTAAAAGGT
ATTGATTTTAAAGAAGATGGAAACATTCTTGGACACAAATTGGAATACAACTATAACTCACACAATGTAT
ACATCATGGCAGACAAACAAAAGAATGGAATCAAAGTTAACTTCAAAATTAGACACAACATTGAAGATG
GAAGCGTTCAACTAGCAGACCATTATCAACAAAATACTCCAATTGGCGATGGCCCTGTCCTTTTACCAGA
CAACCATTACCT

Table S1
Apiaries and number of hives involved in the experiment

Apiary Code	Location	Number of hives for each treatment			Hives excluded from the analyses
		dsT	gfpC	sucC	
TV2	Volpago (TV)	4	4	2	1 sucC hive robbed
TV4	Varago (TV)	5	2	3	
VE1	Ceggia (VE)	5	2	3	1 gfpC hive orphan
VE3	Marcon (VE)	5	2	3	1 sucC hive with anomalous infestation dynamics
PD1	Casalserugo (PD)	5	2	3	All 10 hives because of a mosquito treatment
TV6	Maserada (TV)	Hives dedicated to the collection of *Varroa* mites and adult bees			
